# Characteristics, costs, and outcomes associated with central-line–associated bloodstream infection and hospital-onset bacteremia and fungemia in US hospitals

**DOI:** 10.1017/ice.2023.132

**Published:** 2023-12

**Authors:** Kalvin C. Yu, Molly Jung, ChinEn Ai

**Affiliations:** Becton, Dickinson and Company, Franklin Lakes, New Jersey

## Abstract

**Objectives:**

To compare characteristics and outcomes associated with central-line–associated bloodstream infections (CLABSIs) and electronic health record–determined hospital-onset bacteremia and fungemia (HOB) cases in hospitalized US adults.

**Methods:**

We conducted a retrospective observational study of patients in 41 acute-care hospitals. CLABSI cases were defined as those reported to the National Healthcare Safety Network (NHSN). HOB was defined as a positive blood culture with an eligible bloodstream organism collected during the hospital-onset period (ie, on or after day 4). We evaluated patient characteristics, other positive cultures (urine, respiratory, or skin and soft-tissue), and microorganisms in a cross-sectional analysis cohort. We explored adjusted patient outcomes [length of stay (LOS), hospital cost, and mortality] in a 1:5 case-matched cohort.

**Results:**

The cross-sectional analysis included 403 patients with NHSN-reportable CLABSIs and 1,574 with non-CLABSI HOB. A positive non-bloodstream culture with the same microorganism as in the bloodstream was reported in 9.2% of CLABSI patients and 32.0% of non-CLABSI HOB patients, most commonly urine or respiratory cultures. Coagulase-negative staphylococci and Enterobacteriaceae were the most common microorganisms in CLABSI and non-CLABSI HOB cases, respectively. In case-matched analyses, CLABSIs and non-CLABSI HOB, separately or combined, were associated with significantly longer LOS [difference, 12.1–17.4 days depending on intensive care unit (ICU) status], higher costs (by $25,207–$55,001 per admission), and a >3.5-fold increased risk of mortality in patients with an ICU encounter.

**Conclusions:**

CLABSI and non-CLABSI HOB cases are associated with significant increases in morbidity, mortality, and cost. Our data may help inform prevention and management of bloodstream infections.

Hospital-onset bloodstream infections (BSIs) can compromise patient health and increase the burden on healthcare systems.^1,2^ Improvement in rates of central-line–associated BSIs (CLABSIs) reportable to the National Healthcare Safety Network (NHSN) of the Centers for Disease Control and Prevention (CDC) has inspired reporting to move beyond infections with a central line and a positive culture for an eligible BSI pathogen not related to an infection at another site.^3^ More importantly, concerns about the reliability of CLABSI designations and recognition that CLABSIs account for only a small proportion of hospital-onset BSIs have led to the proposed hospital-onset bacteremia and fungemia (HOB) quality metric.^4–6^ This suggestion passed the National Quality Forum (NQF) Patient Safety Committee review in early 2023 and is being considered by the Centers for Medicare & Medicaid Services (CMS) as a reportable metric.^7^ In addition to providing a more inclusive measure of hospital BSI sources, an HOB metric could be standardized and risk-adjusted using the electronic health record (EHR), thereby eliminating the subjectivity associated with central-line attributions.^8^

Although some studies have explored costs associated with CLABSIs,^2,9^ less is known about the impact of non-CLABSI HOB on hospital outcomes. In this study, we analyzed characteristics, related positive non–blood-culture sites, costs, and patient outcomes associated with CLABSI, non-CLABSI HOB, and all HOB in US hospitals.

## Methods

### Study design and population

We conducted a retrospective observational study of patients in 41 acute-care hospitals in the BD Insights Research and Database (Becton, Dickinson and Company, Franklin Lakes, NJ), which contains electronically captured data encompassing pharmacy, laboratory, administrative data, patient demographics, and admission, discharge, and transfer data feeds.^8,10–12^ The distribution of hospitals in this database is similar to the hospital distribution in the United States as a whole.^12^ Included patients were aged ≥18 years and had been admitted between October 2015 and June 2019. The study was approved as involving use of a limited retrospective data set for an epidemiology study, exempted from consent by the New England Institutional Review Board/ Human Subjects Research Committee (Wellesley, MA), and conducted in compliance with Health Insurance Portability and Accountability Act requirements.

Our study included 2 different analyses: (1) Ecologic cross-sectional comparison of patient characteristics, including other positive cultures and associated microorganisms, for CLABSI and non-CLABSI HOB, and (2) Case-matched analysis of outcomes in patients with CLABSI or non-CLABSI HOB compared with a control population of patients who did not have a BSI. In the case-matched analysis, 3 steps were used to select a comparable control population. (1) Admissions with potential infections (defined as antibiotic duration ≥72 hours, EHR alert for potential infection, or infection diagnosis-related group) were excluded from the control group (Supplementary Table S1 online). (2) Controls and non-CLABSI HOB cases were limited to the same characteristics as the CLABSI case population: age range, major diagnostic code, and *International Classification of Disease Tenth Revision* procedure code system (ICD-10-PCS) code (see Supplementary Material S1 and Table S1 online for details). (3) Cases were matched 1:5 to controls based on ICU status and primary ICD-10-PCS code. The same control group was used for both CLABSI and non-CLABSI HOB to allow comparisons to CLABSI, a reportable infection familiar to clinicians, and was based on methods published for analyses of the costs associated with catheter-associated urinary tract infection (CAUTI).^13^

### Definitions

CLABSI cases were based on CLABSI events reported to the NHSN, which require a laboratory-confirmed BSI in patients with a central line present on the day of the event or before and a BSI organism not related to infection at another site.^3^ Non-CLABSI HOB cases were defined by data available in EHR based on a positive blood culture collected within the hospital-onset period (on or after day 4 of hospitalization) for an eligible BSI organism as defined by the NHSN^3^ (Supplementary Table S2 online) that was not present during the community-onset period. This definition was chosen to match the proposed CDC HOB definition at the time of writing.

### Variables and outcomes

Variables evaluated included associated sites of infection, defined as positive respiratory, urinary, or skin and soft-tissue cultures with the same microorganism species identified in blood that were collected between 2 days prior to and 4 days after the positive blood culture; specified major hospital-acquired infections (HAIs) as reported to the NHSN (CAUTI and surgical site infections [SSI]); intensive care unit (ICU) status; and associated microorganisms. Microorganism identification was based on reports from local laboratory facilities and was performed at the species level. Outcomes included length of stay (LOS), hospital cost per admission, in-hospital mortality rates, and 30-day readmission rates.

### Statistical analysis

The cross-sectional epidemiologic analyses utilized descriptive data. For the case-matched analyses, we used Poisson regression for LOS, γ regression for total cost, and binomial regression for in-hospital mortality and 30-day readmission rates. Models were adjusted for age, sex, Acute Laboratory Risk of Mortality Score (AlaRMS, an EHR-derived comorbidity measure),^10,14–16^ and hospital-level variables (payor, staffed bed size, teaching status, and urban/rural location). All analyses were conducted using R software version 4.1.2 software (R Foundation for Statistical Computing, Vienna, Austria) with R Studio (Boston, MA).

## Results

### Characteristics of CLABSI and non-CLABSI HOB admissions

Of 756,637 total admissions at 41 clinical facilities, 403 (0.05%) had NHSN-reported CLABSI. Two hundred eighty-three (70%) of these admissions also met the definition for HOB used in this study; the remaining 30% had CLABSI with positive cultures for commensal organisms not on the CDC BSI pathogen list (Supplementary Table S2 online). In total, 1,574 admissions (0.21% of total admissions) did not have CLABSI but met the study definition for HOB; this group was designated as “non-CLABSI HOB.” Patient characteristics for CLABSI and non-CLABSI HOB were generally comparable, but the rate of ICU encounters during the hospital admission was higher in the CLABSI subgroup compared with the non-CLABSI HOB subgroup: 73.4% vs 62.1%, respectively (Table 1).

**Table 1.** Characteristics of Patients by CLABSI or Non-CLABSI HOB Status

Characteristic	Ecologic Cross-Sectional Comparison, No. (%)^a^		Case-Matched Analyses, No. (%)^a^			
	CLABSI	Non-CLABSI HOB	Control	CLABSI	Non-CLABSI HOB	All HOB Including CLABSIs
No.	403	1,574	1745	349	1,172	1,521
**Age group, y**						
18–40	59 (14.6)	219 (13.9)	201 (11.5)	49 (14.0)	146 (12.5)	195 (12.8)
41–64	185 (45.9)	652 (41.4)	741 (42.5)	157 (45.0)	499 (42.6)	656 (43.1)
65–80	132 (32.8)	524 (33.3)	589 (33.8)	116 (33.2)	398 (34.0)	514 (33.8)
>80	27 (6.7)	179 (11.4)	214 (12.3)	27 (7.7)	129 (11.0)	156 (10.3)
**Sex**						
Male	209 (51.9)	905 (57.5)	939 (53.8)	182 (52.1)	669 (57.1)	851 (56.0)
Female	194 (48.1)	669 (42.5)	806 (46.2)	167 (47.9)	503 (42.9)	670 (44.0)
**ALaRMS**						
Mean (SD)	60.3 (25.4)	56.4 (23.1)	49.1 (22.1)	61.6 (25.5)	58.2 (23.0)	59.0 (23.7)
Median [IQR]	58.0 [43.0–78.0]	55.0 [41.0–71.0]	46.0 [34.0–62.0]	59.0 [44.5–79.0]	57.0 [43.0–72.0]	57.0 [43.0–73.0]
Missing	2 (0.5)	13 (0.8)	17 (1.0)	2 (0.6)	6 (0.5)	8 (0.5)
**ICU status during admission**						
No	105 (26.1)	592 (37.6)	395 (22.6)	79 (22.6)	391 (33.4)	470 (30.9)
Yes	296 (73.4)	977 (62.1)	1,350 (77.4)	270 (77.4)	781 (66.6)	1,051 (69.1)
Missing	2 (0.5)	5 (0.3)	0	0	0	0
**Payor**						
Medicaid	60 (14.9)	213 (13.5)	157 (9.0)	49 (14.0)	138 (11.8)	187 (12.3)
Medicare	218 (54.1)	891 (56.6)	987 (56.6)	195 (55.9)	685 (58.4)	880 (57.9)
Private	98 (24.3)	347 (22.0)	466 (26.7)	78 (22.3)	256 (21.8)	334 (22.0)
Uninsured	17 (4.2)	63 (4.0)	80 (4.6)	17 (4.9)	51 (4.4)	68 (4.5)
Other	10 (2.5)	58 (3.7)	51 (2.9)	10 (2.9)	40 (3.4)	50 (3.3)
Missing	0 (0)	2 (0.1)	4 (0.2)	0 (0)	2 (0.2)	2 (0.1)
**Staffed bed size**						
<100	6 (1.5)	41 (2.6)	42 (2.4)	5 (1.4)	31 (2.6)	36 (2.4)
100–300	80 (19.9)	281 (17.9)	475 (27.2)	74 (21.2)	218 (18.6)	292 (19.2)
>300	317 (78.7)	1252 (79.5)	1228 (70.4)	270 (77.4)	923 (78.8)	1193 (78.4)
**Teaching status**						
Nonteaching	96 (23.8)	384 (24.4)	592 (33.9)	92 (26.4)	306 (26.1)	398 (26.2)
Teaching	307 (76.2)	1190 (75.6)	1153 (66.1)	257 (73.6)	866 (73.9)	1123 (73.8)
**Urban status**						
Rural	64 (15.9)	193 (12.3)	248 (14.2)	60 (17.2)	153 (13.1)	213 (14.0)
Urban	339 (84.1)	1381 (87.7)	1497 (85.8)	289 (82.8)	1019 (86.9)	1308 (86.0)

Note. ALaRMS, Acute Laboratory Risk of Mortality Score; CLABSI, central-line–associated bloodstream infection; HOB, hospital-onset bacteremia; IQR, interquartile range; ICU, intensive care unit; SD, standard deviation.

a Units unless otherwise specified.

### Association of CLABSI and non-CLABSI HOB with sites of infection and microorganisms

We evaluated whether patients with CLABSI or non-CLABSI HOB also had positive cultures for the bloodstream microorganism at other specified sites (urine, respiratory, and skin and soft-tissue) (Table 2). As might be expected from the definition of CLABSI, only 37 of 403 patients with CLABSI (9.2%) had a positive culture at another site with the same microorganism found in the blood culture. In contrast, 504 (32%) of 1,574 non-CLABSI HOB patients had a  microorganism at another site that was the same as the HOB-defining blood culture, most commonly in urine (12.7%) or respiratory (10.4%) cultures (Table 2). Analyses by ICU status showed markedly higher rates of positive respiratory cultures with the same microorganism in non-CLABSI HOB admissions with ICU encounters (15.6%) compared with non-ICU encounters (1.9%) (Supplementary Table S3 online). Rates of positive urinary and skin and soft-tissue cultures were similar for ICU versus non-ICU admissions. Specified HAIs (CAUTI or SSI) with the same organism were not identified in patients with CLABSI but were reported in 2.7% of patients with non-CLABSI HOB (Supplementary Table S4 online).

**Table 2.** Association of HOB With Other Positive Cultures From Specified Sites as Determined by Identification of the Same Microorganism From Both Sources

Culture Site	CLABSI (N=403), No. (%)	Non-CLABSI HOB (N=1,574), No. (%)	All HOB including CLABSI (N=1,977), No. (%)
None of the below	366 (90.8)	1,070 (68.0)	1,436 (72.6)
Urine only	19 (4.7)	200 (12.7)	219 (11.1)
Respiratory only	11 (2.7)	163 (10.4)	174 (8.8)
Skin/soft tissue only	5 (1.2)	93 (5.9)	98 (5.0)
Urine and respiratory	0	19 (1.2)	19 (1.0)
Skin/soft tissue and respiratory	0	15 (1.0)	15 (0.8)
Urine and skin/soft tissue	2 (0.5)	10 (0.6)	12 (0.6)
Urine, skin/soft tissue, and respiratory	0	4 (0.3)	4 (0.2)

Note. CLABSI, central-line–associated bloodstream infection; HOB, hospital-onset bacteremia; SSTI, skin and soft-tissue infection. Categories for positive culture sites are mutually exclusive.

For CLABSI admissions, the most common bloodstream microorganism was coagulase-negative staphylococci (CoNS; 20.6%) followed by Enterobacteriaceae (16.6%) and *Enterococcus* spp (15.9%) (Table 3). For non-CLABSI HOB admissions, the most common bloodstream microorganisms were Enterobacteriaceae (36.5%) followed by *Staphylococcus aureus* (25.6%) and *Enterococcus* spp (15.8%). CoNS were excluded from eligible pathogens for non-CLABSI HOB.^8^

**Table 3.** Microorganisms Identified in CLABSI and Non-CLABSI HOB Admissions

Microorganism^a^	CLABSI (N=403), No. (%)	Non-CLABSI HOB (N=1,574), No. (%)	All HOB including CLABSI (N=1,977), No. (%)
Enterobacteriaceae	67 (16.6)	575 (36.5)	642 (32.5)
*S. aureus*	50 (12.4)	403 (25.6)	453 (22.9)
Enterococcus spp.	64 (15.9)	248 (15.8)	312 (15.8)
Environmental GNB	34 (8.4)	179 (11.4)	213 (10.8)
*Candida albicans* and *C. auris*	52 (12.9)	123 (7.8)	175 (8.9)
Other *Candida* spp^a^	53 (13.2)	117 (7.4)	170 (8.6)
CoNS	83 (20.6)	0	83 (4.2)
Other GPB	33 (8.2)	0	33 (1.7)
Other GNB	18 (4.4)	0	17 (0.9)
Other commensal	17 (4.2)	0	17 (0.9)
No microorganism	12 (3.0)	0	12 (0.6)

Note. CLABSI, central-line–associated bloodstream infection; CoNS, coagulase-negative staphylococci; GNB, gram-negative bacteria; GNP, gram-positive bacteria; HOB, hospital-onset bacteremia.

a See Supplementary Table S2 (online) for a full list of included microorganisms.

For patients who had positive urine cultures with the same microorganism as the HOB, Enterobacteriaceae was the most common microorganism (61% of positive urine cultures in all HOB admissions). *S. aureus* was the most common shared microorganism present in respiratory (42%) and skin/soft tissue (53%) cultures (Supplementary Table S5). *Enterococcus* spp, environmental gram-negative bacteria (including *Acinetobacter* spp), and fungi and/or yeast (ie, *Candida* spp) were also recorded in both blood and other specified culture sources (see Supplementary Table S2 online for specific microorganisms in each category). CoNS, other commensals, other gram-positive bacteria, and other gram-negative bacteria were found in blood cultures only and not shared with other sites of infection, with 1 exception (ie, a CLABSI admission with a positive respiratory culture for other gram-negative bacteria) (Supplementary Table S5 online).

### Case-matched cohort

The case-matched cohort included 349 admissions with CLABSI, 1,172 admissions with non-CLABSI HOB, and 1,745 CLABSI-matched control admissions. Cases and controls had generally similar characteristics except for ALaRMS values (ie, a comorbidity score that estimates likelihood of death within an admission)^10,14–16^ (Table 1). This variable was adjusted for in the final model. Admissions with an ICU encounter during the specified visit included 270 CLABSI cases, 781 non-CLABSI HOB cases, and 1,350 controls. Admission with non-ICU encounters included 79 CLABSI cases, 391 non-CLABSI HOB cases, and 395 controls. Subgroup characteristics by ICU status are shown in Supplementary Table S6 (online).

### Outcomes associated with CLABSI and non-CLABSI HOB in case-matched analyses by ICU status

In adjusted regression analyses, CLABSI and non-CLABSI HOB cases were associated with significantly longer hospital stays compared with the control cohort. The mean differences were 15.6 and 12.1 days, respectively, for admissions with non-ICU encounters; 17.4 and 14.9 days for admissions with an ICU encounter; and 16.8 and 13.8 days for combined ICU/non-ICU admissions. Hospital costs were also higher for CLABSI and non-CLABSI HOB cases compared with controls: mean differences of $32,759 and $25,207 per admission for non-ICU encounters; $55,001 and $42,095, respectively, for ICU encounters and $49,400 and $35,310, respectively, for all admissions (Table 4 and Supplementary Table S7 online). The LOS for CLABSI was slightly higher than for non-CLABSI HOB cases. The adjusted means were 19.2 versus 15.9 days, respectively, for admissions with non-ICU encounters and 22.5 vs 19.6 days, respectively, for admissions with ICU encounters. Total hospital costs for CLABSI were also higher than for non-CLABSI HOB: $42,201 versus $34,243, respectively, for non-ICU encounters and $70,407 vs $57,262, respectively, for ICU encounters (Table 4).

**Table 4.** Outcomes for BSI Cases and Controls^a^

Outcome	Non-ICU						ICU					
	CLABSI Analyses		Non-CLABSI HOB Analyses		All HOB Including CLABSI Analyses		CLABSI Analyses		Non-CLABSI HOB Analyses		All HOB Including CLABSI Analyses	
	Control	Cases	Control	Cases	Control	Cases	Control	Cases	Control	Cases	Control	Cases
No.	395	79	395	391	395	470	1350	270	1350	781	1350	1051
**Length of stay**												
Adjusted mean days (95% CI)^b^	3.6 (3.0–4.4)	19.2 (15.8–23.3)	3.8 (3.3– 4.3)	15.9 (13.9–18.1)	3.7 (3.3–4.2)	16.2 (14.4–18.1)	5.1 (4.6–5.7)	22.5 (20.0–25.2)	4.7 (4.2–5.3)	19.6 (17.5–22.0)	4.8 (4.3–5.3)	20.4 (18.3–22.7)
Difference (case – control)	15.6 *P* <.001		12.1 *P* <.001		12.5 *P* <.001		17.4 *P* <.001		14.9 *P* <.001		15.6 *P* <0.001	
**Total hospital cost**												
Adjusted mean USD (95% CI)^b^	$9,442 (7,061– 12,626)	$42,201 (30,882– 57,669)	$9,036 (6,574–12,091)	$34,243 (25,479–46,021)	$9,268 (7,073–12,145)	$36,335 (27,745–47,584)	$15,406 (12,123–19,578)	$70,407 (54,717–90,596)	$15,168 (11,999–19,173)	$57,262 (45,185–72,568)	$15,267 (12,123–19,227)	$60,638 (47,875–76,122)
Difference (case – control)	$32,759 *P* <.001		$25,207 *P* <.001		$27,067 *P* <.001		$55,001 *P* <.001		$42,095 *P* <.001		$45,101 *P* <.001	
**Mortality rate**												
Unadjusted, no. (%)	5 (1.3)	12 (15.2)	5 (1.3)	46 (11.8)	5 (1.3)	58 (12.3)	129 (9.6)	97 (35.9)	129 (9.6)	274 (35.1)	129 (9.6)	371 (35.3)
Adjusted % (95% CI)^b^	Too low to accurately calculate		Too low to accurately calculate		Too low to accurately calculate		3% (1–7)	11% (5–23)	6% (4–9)	21% (14–30)	6% (4–9)	20% (14–28)
Relative risk (cases vs controls)	Too low to accurately calculate		Too low to accurately calculate		Too low to accurately calculate		3.52 *P*<.001		3.51 *P* <.001		3.58 *P* <.001	
**30-day readmission rate**												
Unadjusted, no. (%)	67 (17.0)	26 (32.9)	67 (17.0)	94 (24.0)	67 (17.0)	120 (25.5)	165 (12.2)	34 (12.6)	165 (12.2)	96 (12.3)	165 (12.2)	130 (12.4)
Adjusted % (95% CI)^b^	13 (6–26)	26 (12–47)	12 (6–24)	17 (8–32)	13 (7–23)	21 (12–33)	12 (8–18)	14 (8–23)	13 (10–19)	14 (10–20)	13 (10–18)	14 (10–20)
Relative risk (cases vs controls)	2.07 *P*=.003		1.45 *P*=.024		1.59 *P*=.003		1.16 *P*=.432		1.06 *P*=.647		1.10 *P*=.420	

Note. ALaRMS, Acute Laboratory Risk of Mortality Score; BSI, bloodstream infection; CI, confidence interval; CLABSI, central-line–associated bloodstream infection; HOB, hospital-onset bacteremia; ICU, intensive care unit; LOS, length of stay.

a Control data for CLABSI vary slightly from control data for non-CLABSI HOB due to differences in adjustments based on cohort composition. “Non-ICU” and “ICU” refer to ICU admission during the admission associated with the current hospitalization.

b Adjusted for age, sex, ALaRMS value, and hospital-level variables (payer, staffed bed size, teaching status, and urban/rural location).

Unadjusted in-hospital mortality rates were significantly higher for HOB cases versus controls overall: 31.3% for CLABSI; 28.3% for non-CLABSI HOB; and 8.3% for controls (Supplementary Table S7) and when stratified by ICU status (Table 4). In combined ICU and non-ICU analyses, CLABSI and non-CLABSI HOB were associated with significantly increased relative mortality risks of 3.77 and 3.20, respectively (Supplementary Table S7 online). Mortality rates for admissions with non-ICU encounters were too low to accurately calculate relative risk. For admissions with an ICU encounter, CLABSI and non-CLABSI HOB were associated with relative mortality risks of 3.52 and 3.51, respectively (Table 4). The 30-day readmission rates were significantly higher for CLABSI and non-CLABSI HOB cases overall (relative risk, 1.41 and 1.28, respectively) and for admissions with non-ICU encounters (relative risk, 2.07 and 1.45 for CLABSI and non-CLABSI HOB admissions, respectively). (Supplementary Table S7 online). The 30-day readmission rates in cases with ICU encounters did not differ significantly from controls (Table 4).

## Discussion

In this multicenter database encompassing almost 800,000 hospitalized adult patients, there were ∼4 times as many non-CLABSI HOB cases as CLABSI cases (1,574 vs 403) using a conservative EHR-based case definition that only included the first positive blood culture with a CDC-designated BSI pathogen.^8^ Compared with matched controls, both CLABSIs and non-CLABSI HOB cases were associated with increases in LOS and significantly higher hospital costs, mortality rates, and 30-day readmission rates. Rock et al^4^ reported that among ICU patients, changes in HOB rates are highly associated with changes in CLABSI rates. These data suggest that it is feasible for HOB to serve as a quality metric indicator while including more BSI patients than the current CLABSI-reportable events, and that such a change may be consequential. Thus, we highlight 2 key points: (1) the extra cost of care required for CLABSI and non-CLABSI HOB cases and (2) the hospital stay, mortality burden, and readmission risk related to both. Reducing HOB and/or improving time to definitive therapy through the existing required infrastructures of infection prevention and antimicrobial stewardship programs has the potential to improve patient safety.^17^

Overall admission characteristics for CLABSI and non-CLABSI HOB were generally similar, although the CLABSI cohort had a higher rate of ICU encounters (73.4% vs 62.1%). Probably due to criteria used for the CLABSI designation, which exclude cases related to another infection,^3^ non-CLABSI HOB cases were more likely to have another positive culture, most commonly urine or respiratory, and to be associated with other reportable HAIs (CAUTI or SSI). Although our study was not designed to definitively evaluate the sources of HOB, we found that 27.4% of all HOB admissions had other positive cultures with the same microorganism found in the blood culture within the specified time frame. Notably, we evaluated urinary, respiratory, and skin and soft-tissue cultures, but we did not include other sources potentially associated with HOB, including the gastrointestinal system,^6,18,19^ or poorly or nondocumented sources such as peripheral intravenous devices.

The designation of CLABSI is predicated on excluding temporal and clinical relationships to another culture site with the same pathogen. It is often difficult to differentiate secondary versus primary infection; this distinction is no longer relevant for HOB. Removal of the requirement for attributing primary and secondary sources of infection may refocus attention on understanding factors driving HOB, which in turn may redirect emphasis to prevention or timely treatment of infections that may be a source of HOB, essentially bringing patient care back as the focus rather than whether the event is reportable or not.

Enterobacteriaceae was the most common bloodstream microorganism for non-CLABSI HOB, whereas CoNS was the most common bloodstream microorganism for CLABSI. Other common microorganisms included *S. aureus* and *Enterococcus* spp. These data are consistent with a recent analysis of pathogens associated with BSI in Ontario, which identified *Escherichia coli* as the most common pathogen.^20^ However, other studies have found a larger contribution by *S. aureus* and CoNS and a diminished role for Enterobacteriaceae.^6,19^ The reasons for the different pathogen distributions among studies may be due to differences in patient populations and in case study parameters. For example, we excluded CDC commensal microorganisms from the HOB definition to be consistent with a recently published paper;^8^ however, commensals can be true pathogens depending on the number, proximity, and clinical interpretation of positive blood cultures.^21,22^

Our data support and update prior reports on the burden of HOB in hospitalized patients. The ∼30% mortality rate of HOB in our study is generally comparable to HOB mortality rates reported in other US hospitals^23,24^ and in Denmark,^25^ and the mortality rate of 35.3% in patients with an ICU encounter is similar to the 36.6% reported in ICU admissions in France.^26^ The similarity of these data suggest that challenges in managing these infections transcend individual healthcare systems and case-mix differences; these infections remain a ubiquitous problem in healthcare that warrants a larger spotlight. The HOB LOS and costs reported here provide an important update to earlier US studies encompassing smaller numbers of hospitals^23,24^ and focused on estimates for CLABSI costs.^2,9^ Significant increases in 30-day readmission rates compared with controls were observed for patients who did not have an ICU encounter, but not those with ICU admissions, perhaps suggesting that the complexity of managing HOB may impact the stability of patients at the time of discharge from a regular hospital ward.

This study had several limitations. The definition of HOB used by the NHSN may undergo further iteration as more is learned about its use in infection prevention. We evaluated non-CLABSI HOB to provide insights into the additional burden of BSI cases not captured by reportable CLABSI events. However, as noted earlier, only first positive blood cultures were considered eligible for HOB designation, so HOB prevalence may have been underestimated. For our exploration of associated positive cultures, microorganism identification was based on species only. More frequent culturing in ICU patients may have led to increased identification of microorganisms compared with non-ICU patients. In case-matched analyses, controls were matched to CLABSI cases but not to non-CLABSI HOB cases; however, analyses were adjusted to make outcome comparisons more similar. As with any case-matched analyses, we acknowledge that differential outcomes are highly dependent on the control population. However, sensitivity analyses in which ICU status was not considered as a matching criterion resulted in similar differences in outcomes. Finally, sources of HOB were assigned to categories that could be feasibly extracted from EHR. For example, quantification of secondary HOBs associated with peripheral IV lines was beyond the scope of this analysis. Future studies will be needed to delineate a more granular source attribution list for HOB to facilitate infection preventionist efforts to mitigate HOB events.

The findings from this study document the extensive burden associated with non-CLABSI HOB in hospitalized US patients. Expanding reporting metrics to include non-CLABSI HOB will help provide a more comprehensive view of BSI control in hospitals and may allow new approaches to reducing the rate of these consequential infections. As healthcare systems continue to adjust to financial ramifications of the COVID-19 pandemic,^27,28^ demonstration of the economic and patient burdens of HOB will be important in vetting this outcome as a reportable hospital quality metric. In the process, this information will provide insights into opportunities for improved patient safety and reduced hospital costs beyond those associated with NHSN-reportable CLABSI events.^2^

## Supplementary material

For supplementary material accompanying this paper visit https://doi.org/10.1017/ice.2023.132. click here to view supplementary material
