# Supplementary material for: Characteristics, costs, and outcomes associated with central-line–associated bloodstream infection and hospital-onset bacteremia and fungemia in US hospitals
Source: Infect Control Hosp Epidemiol. 2023 Jul 10;44(12):1920–6. doi: 10.1017/ice.2023.132 (PMC10755163; doi:10.1017/ice.2023.132)
Supplement: Supplementary file 1 [file S0899823X23001320sup.zip › S0899823X23001320sup002.docx]

**Supplementary Information**

**Characteristics, costs, and outcomes associated with central line-associated bloodstream infection and hospital-onset bacteremia and fungemia in US hospitals**

Kalvin C. Yu MD^1^, Molly Jung PhD^1^, ChinEn Ai MPH^1^

^1^Becton, Dickinson and Company, 1 Becton Drive, Franklin Lakes, New Jersey, USA

**Section S1.** Matching for case-matched analysis

**Table S1.** Analytic cohort matching description

**Table S2.**  Microorganism definitions. [see separate Excel spreadsheet]

**Table S3**. Association of HOB with other specified sites of infection and specified hospital-acquired infections as determined by identification of the same pathogen species from both sources by ICU status.

**Table S4**. Association of HOB with other positive cultures from specified hospital-acquired infections as determined by identification of the same microorganism from both sources.

**Table S5.** Association of microorganisms in CLABSI or non-CLABSI HOB with the same microorganism from another specified source (urine, respiratory, or skin/soft tissue).

**Table S6.** Patient and hospital characteristics for matched cases and controls stratified by ICU admission at any point during the specified hospital visit.

**Table S7**. Outcomes for BSI cases and controls.

**Section S1.** Matching for case-matched analysis

For matching of cases to controls (1:5), (1) admissions with potential infections (defined as antibiotic duration ≥72 hours, EHR alert for potential infection, or infection diagnosis-related group) were excluded from the control group (Supplementary Table S1); (2) controls and non-CLABSI HOB cases were limited to the same characteristics as the CLABSI case population including age range, major diagnostic code, and International Classification of Disease 10^th^ revision procedure code system (ICD-10-PCS) code. We matched 5 controls to each case based on the same ICU status and the Agency for Healthcare Research and Quality Clinical Classification Software (CCS) single-level procedure category of the primary ICD-10-PCS procedure code. If cases did not have applicable controls in step 1 and 2, they were excluded (54/403 [13%] CLABSI cases and 402/1574 [26%] of non-CLABSI HOB cases were excluded from the case-matched analyses). We used the same control group for CLABSI and non-CLABSI-HOB to allow comparisons of the incremental burden associated with cases compared with non-BSI controls. Multivariable adjusted regression models were adjusted for patient-level characteristics (age, sex, and AlaRMS comorbidity score) and hospital-level characteristics (payer, staffed bed size, teaching status, and urbanicity).

**Supplementary Table S1.** Analytic cohort matching description

| **Inclusion/Exclusion criteria** | **Number of hospitals** | **Number in patient cohort** | **Number of excluded patients** | **Number of patients with CLABSI** | **% of cohort** | **Number of patients with non-CLABSI HOB** | **% of cohort** | **Number of control patients** | **% of cohort** |
| --- | --- | --- | --- | --- | --- | --- | --- | --- | --- |
| Total hospitals^a^, acute care admissions with patient age ≥ 18 years^b^ | 85 | 1,149,448 |  |  |  |  |  |  |  |
| At least 1 CLABSI and reporting of blood infection to NHSN of blood NIM | 41 | 756,637 | 392,811 |  |  |  |  |  |  |
| Exclude if LOS >365 days | 41 | 645,315 | 111,322 |  |  |  |  |  |  |
| Cross-sectional cohort | 41 | 645,315 |  | 403 | 0.06% | 1,574 | 0.244% | 643,338 |  |
| Exclude controls with potential infection^c^ | 41 | 461,888 | 183,427 | 403 | 100.0% | 1,574 | 100.0% | 459,911 | 71.5% |
| Exclude if missing ICU status | 41 | 453,481 | 8,407 | 401 | 99.5% | 1,569 | 99.7% | 451,511 | 70.2% |
| Exclude if age of control is outside of cases | 41 | 453,481 | - | 401 | 99.5% | 1,569 | 99.7% | 451,511 | 70.2% |
| Exclude if control MDC does not match case | 41 | 358,278 | 95,203 | 401 | 99.5% | 1,511 | 96.0% | 356,366 | 55.4% |
| Exclude if primary PCS does not match case | 39 | 164,816 | 193,462 | 358 | 88.8% | 1,200 | 76.2% | 163,258 | 25.4% |
| Case control cohort (1:5 match between CLABSI cases and controls) | 39 | 3,266 | 161,550 | 349 | 86.6% | 1,172 | 74.5% | 1,745 | 0.3% |

^a^All sites with appropriate data (defined as at least 3 months of consecutive data with pharmacy, general laboratory, financial, and microbiology data available with a high completion rate)

^b^Acute care hospitals, excluding children’s long term care facilities and specialty hospitals

^c^Defined as antibiotic duration ≥72 hours, electronic health record alert for potential infection, or infection diagnosis-related group.

Note. CLABSI, central-line associated bloodstream infection; HOB, hospital-onset bacteremia; ICU, intensive care unit; LOS, length of stay; MDC, major diagnostic code; NHSN, National Health Safety Network; NIM, nosocomial infection marker; PCS, procedure coding system.

**Supplementary Table S2.**  Microorganism definitions. [see separate Excel spreadsheet]

**Supplementary Table S3**. Association of HOB with other specified sites of infection as determined by identification of the same pathogen species from both sources by ICU status. Data are presented as n (%).

| **Other sites of infection^c^** | **CLABSI^a^** | | **Non-CLABSI HOB^b^** | | **All HOB including CLABSI** | |
| --- | --- | --- | --- | --- | --- | --- |
|  | **ICU**  **(n=296)** | **No ICU**  **(n=105)** | **ICU**  **(n=977)** | **No ICU**  **(n=592)** | **ICU**  **(n=1273)** | **No ICU**  **(n=697)** |
| None of the below | 268 (90.5%) | 96 (91.4%) | 614 (62.8%) | 452 (76.4%) | 882 (69.3%) | 548 (78.6%) |
| Urine only | 15 (5.1%) | 4 (3.8%) | 118 (12.1%) | 82 (13.9%) | 133 (10.4%) | 86 (12.3%) |
| Respiratory only | 10 (3.4%) | 1 (1.0%) | 152 (15.6%) | 11 (1.9%) | 162 (12.7%) | 12 (1.7%) |
| Skin/soft tissue only | 2 (0.7%) | 3 (2.9%) | 54 (5.5%) | 39 (6.6%) | 56 (4.4%) | 42 (6.0%) |
| Urine and respiratory | 0 | 0 | 15 (1.5%) | 3 (0.5%) | 15 (1.2%) | 3 (0.4%) |
| Urine and skin/soft tissue | 1 (0.3%) | 1 (1.0%) | 8 (0.8) | 2 (0.3%) | 9 (0.7%) | 3 (0.4%) |
| Skin/soft tissue and respiratory | 0 | 0 | 12 (1.2%) | 3 (0.5%) | 12 (0.9% | 3 (0.4%) |
| Urine, respiratory, and skin/soft tissue | 0 | 0 | 4 (0.4%) | 0 | 4 (0.3%) | 0 |

^a^ICU status was unknown for 2 admissions

^b^ICU status was unknown for 5 admissions

^c^Categories for sites of infection are mutually exclusive

Note. CLABSI, central-line associated bloodstream infection; HOB, hospital-onset bacteremia; ICU, intensive care unit; SSI, surgical site infection.

**Supplementary Table S4**. Association of HOB with other positive cultures from specified HAIs as determined by identification of the same microorganism from both sources. Data are presented as n (%). Categories for HAI are mutually exclusive .

| **HAI** | **CLABSI**  **(N=403)** | **Non-CLABSI HOB**  **(N=1574)** | **All HOB including CLABSI**  **(N=1977)** |
| --- | --- | --- | --- |
| None of the below | 403 (100%) | 1531 (97.3%) | 1934 (97.8%) |
| CAUTI | 0 | 41 (2.6%) | 41 (2.1%) |
| SSI | 0 | 9 (0.6%) | 9 (0.5%) |

Note. CLABSI, central-line associated bloodstream infection; HAI, hospital-acquired infection; HOB, hospital-onset bacteremia; SSI, surgical site infection.

**Supplementary Table S5.** Association of microorganisms in CLABSI or non-CLABSI HOB with the same microorganism from another specified source (urine, respiratory, or skin/soft tissue). Data are presented as n (% of source). Patients could have multiple sources of infection or microorganisms. Percentages may not total to 100 due to rounding.

| **Micro-organism** | **n for CLABSI,**  **non-CLABSI HOB, and all HOB^a^** | **Urine** | | | **Respiratory** | | | **Skin/soft tissue** | | | **No specified non-blood source** | | |
| --- | --- | --- | --- | --- | --- | --- | --- | --- | --- | --- | --- | --- | --- |
|  |  | **CLABSI**  **(n=21)** | **Non-CLABSI HOB**  **(n=233)** | **All HOB^a^**  **(n=254)** | **CLABSI**  **(n=11)** | **Non-CLABSI HOB**  **(n=201)** | **All HOB^a^**  **(n=212)** | **CLABSI**  **(n=7)** | **Non-CLABSI HOB**  **(n=122)** | **All HOB^a^**  **(n=129)** | **CLABSI**  **(n=379)** | **Non-CLABSI HOB**  **(n=1154)** | **All HOB***  **(n=1533)** |
| Enterobac-teriaceae | 67  575  642 | 7 (33%) | 149 (64%) | 156 (61%) | 2 (18%) | 56 (27%) | 58 (27%) | 2 (29%) | 29 (24%) | 31 (24%) | 61 (13%) | 385 (32%) | 446 (27%) |
| *S. aureus* | 50  403  453 | 2 (10%) | 25 (11%) | 27 (11%) | 5 (45%) | 85 (41%) | 90 (42%) | 4 (57%) | 65 (53%) | 69 (53%) | 43 (9%) | 263 (22%) | 306 (19%) |
| Entero-coccus spp. | 64  248  312 | 4 (19%) | 15 (6%) | 19 (7%) | 1 (9%) | 3 (1%) | 4 (2%) | 1 (14%) | 9 (7%) | 10 (8%) | 58 (13%) | 226 (19%) | 284 (17%) |
| Environ-mental GNB^b^ | 18  179  213 | 3 (14%) | 35 (15%) | 38 (15%) | 2 (18%) | 50 (24%) | 52 (24%) | 0 | 13 (11%) | 13 (10%) | 29 (6%) | 102 (9%) | 131 (8%) |
| *Candida albicans* and *Candida auris* | 52  123  175 | 1 (5%) | 3 (1%) | 4 (2%) | 0 | 7 (3%) | 7 (3%) | 0 | 1 (1%) | 1 (1%) | 51 (11%) | 114 (10%) | 165 (10%) |
| Other *Candida* spp^c^ | 53  117  170 | 4 (19%) | 6 (3%) | 10 (4%) | 0 | 4 (2%) | 4 (2%) | 0 | 5 (4%) | 5 (4%) | 50 (11%) | 107 (9%) | 157 (10%) |
| CoNS^d^ | 83  0  83 | 0 | 0 | 0 | 0 | 0 | 0 | 0 | 0 | 0 | 83 (18%) | 0 | 83 (5%) |
| Other GPB^c^ | 33  0  33 | 0 | 0 | 0 | 0 | 0 | 0 | 0 | 0 | 0 | 33 (7%) | 0 | 33 (2%) |
| Other GNB^c^ | 18  0  18 | 0 | 0 | 0 | 1 (9%) | 0 | 1 (9%) | 0 | 0 | 0 | 17 (4%) | 0 | 17 (1%) |
| Other commen-sal^b,d^ | 17  0  17 | 0 | 0 | 0 | 0 | 0 | 0 | 0 | 0 | 0 | 17 (4%) | 0 | 17 (1%) |
| No pathogen specified | 12  0  12 | 0 | 0 | 0 | 0 | 0 | 0 | 0 | 0 | 0 | 12 (3%) | 0 | 12 (1%) |

^a^Including CLABSI

^b^Primarily *Acinetobacter* spp. and *Burkholderia (Pseudomonas)* spp. See Supplementary Table S2 for full list of included microorganisms.

^c^See Supplementary Table S2 for full list of included microorganisms.

^d^Admissions with CoNS or other commensals were excluded from non-CLABSI HOB events

Note. CLABSI, central-line associated bloodstream infection; CoNS, coagulase-negative staphylococci; GNB, Gram-negative bacteria; GPB, Gram-positive bacteria; HOB, hospital-onset bacteremia; Resp, respiratory; spp., species

**Supplementary Table S6.** Patient and hospital characteristics for matched cases and controls stratified by ICU admission at any point during the specified hospital visit.

| **Characteristic** | **No ICU** | | | | **ICU** | | | |
| --- | --- | --- | --- | --- | --- | --- | --- | --- |
|  | **CLABSI** | **Non-CLABSI HOB** | **All HOB Including CLABSI** | **Control** | **CLABSI** | **Non-CLABSI HOB** | **All HOB Including CLABSI** | **Control** |
| N | 79 | 391 | 470 | 395 | 270 | 781 | 1051 | 1350 |
| Age group (years) |  |  |  |  |  |  |  |  |
| 18-40 | 16 (20.3%) | 64 (16.4%) | 80 (17.0%) | 53 (13.4%) | 33 (12.2%) | 82 (10.5%) | 115 (10.9%) | 144 (10.7%) |
| 41-64 | 34 (43.0%) | 160 (40.9%) | 194 (41.3%) | 166 (42.0%) | 123 (45.6%) | 339 (43.4%) | 462 (44.0%) | 550 (40.7%) |
| 65-80 | 23 (29.1%) | 118 (30.2%) | 141 (30.0%) | 123 (31.1%) | 93 (34.4%) | 280 (35.9%) | 373 (35.5%) | 484 (35.9%) |
| >80 | 6 (7.6%) | 49 (12.5%) | 55 (11.7%) | 53 (13.4%) | 21 (7.8%) | 80 (10.2%) | 101 (9.6%) | 172 (12.7%) |
| Sex |  |  |  |  |  |  |  |  |
| Male | 35 (44.3%) | 216 (55.2%) | 251 (53.4%) | 187 (47.3%) | 147 (54.4%) | 453 (58.0%) | 600 (57.1%) | 753 (55.8%) |
| Female | 44 (55.7%) | 175 (44.8%) | 219 (46.6%) | 208 (52.7%) | 123 (45.6%) | 328 (42.0%) | 452 (42.9%) | 597 (44.2%) |
| ALaRMS Score |  |  |  |  |  |  |  | 0 (0%) |
| Mean (SD) | 48.0 (20.1) | 50.3 (20.0) | 49.9 (20.0) | 40.6 (17.1) | 65.6 (25.6) | 62.2 (23.5) | 63.1 (24.1) |  |
| Median (Q1, Q3) | 47.0 [36.0, 60.0] | 51.0 [38.0, 63.0] | 50.0 [38.0, 62.0] | 40.0 [29.0, 51.0] | 62.0 [47.0, 83.0] | 60.0 [45.0, 77.0] | 61.0 [45.0, 79.0] | 52.6 (22.5) |
| Missing | 1 (1.3%) | 3 (0.8%) | 4 (0.9%) | 6 (1.5%) | 1 (0.4%) | 3 (0.4%) | 4 (0.4%) | 50.0 [37.0, 67.0] |
| Payor |  |  |  |  |  |  |  |  |
| Medicaid | 13 (16.5%) | 42 (10.7%) | 55 (11.7%) | 32 (8.1%) | 36 (13.3%) | 96 (12.3%) | 132 (12.6%) | 114 (8.4%) |
| Medicare | 42 (53.2%) | 232 (59.3%) | 274 (58.3%) | 221 (55.9%) | 153 (56.7%) | 453 (58.0%) | 606 (57.7%) | 787 (58.3%) |
| Other | 5 (6.3%) | 9 (2.3%) | 14 (3.0%) | 8 (2.0%) | 5 (1.9%) | 31 (4.0%) | 36 (3.4%) | 53 (3.9%) |
| Private | 16 (20.3%) | 93 (23.8%) | 109 (23.2%) | 117 (29.6%) | 62 (23.0%) | 163 (20.9%) | 225 (21.4%) | 331 (24.5%) |
| Uninsured | 3 (3.8%) | 15 (3.8%) | 18 (3.8%) | 16 (4.1%) | 14 (5.2%) | 36 (4.6%) | 50 (4.8%) | 61 (4.5%) |
| Missing | 0 (0%) | 0 (0%) | 0 (0%) | 1 (0.3%) | 0 (0%) | 2 (0.3%) | 2 (0.2%) | 4 (0.3%) |
| Staffed bed size |  |  |  |  |  |  |  |  |
| <100 | 3 (3.8%) | 9 (2.3%) | 12 (2.6%) | 5 (1.3%) | 2 (0.7%) | 22 (2.8%) | 24 (2.3%) | 41 (3.0%) |
| 100-300 | 16 (20.3%) | 85 (21.7%) | 101 (21.5%) | 115 (29.1%) | 58 (21.5%) | 133 (17.0%) | 191 (18.2%) | 372 (27.6%) |
| >300 | 60 (75.9%) | 297 (76.0%) | 357 (76.0%) | 275 (69.6%) | 210 (77.9%) | 626 (80.2%) | 836 (79.5%) | 937 (69.4%) |
| Teaching status |  |  |  |  |  |  |  |  |
| Non-teaching | 16 (20.3%) | 119 (30.4%) | 135 (28.7%) | 133 (33.7%) | 76 (28.1%) | 187 (23.9%) | 263 (25.0%) | 477 (35.3%) |
| Teaching | 63 (79.7%) | 272 (69.6%) | 335 (71.3%) | 262 (66.3%) | 194 (71.9%) | 594 (76.1%) | 788 (75.0%) | 873 (64.7%) |
| Urban status |  |  |  |  |  |  |  |  |
| Rural | 12 (15.2%) | 46 (11.8%) | 58 (12.3%) | 64 (16.2%) | 48 (17.8%) | 107 (13.7%) | 155 (14.7%) | 202 (15.0%) |
| Urban | 67 (84.8%) | 345 (88.2%) | 412 (87.7%) | 331 (83.8%) | 222 (82.2%) | 674 (86.3%) | 896 (85.3%) | 1148 (85.0%) |

Note. ALaRMS, Acute Laboratory Risk of Mortality Score; CLABSI, central-line associated bloodstream infection; HOB, hospital-onset bacteremia; ICU, intensive care unit; SD, standard deviation.

**Supplementary Table S7**. Outcomes for BSI cases and controls. Control data for CLABSI vary slightly from control data for non-CLABSI HOB due to differences in adjustments based on cohort composition.

| **Outcome** | **CLABSI Analyses** | | **Non-CLABSI HOB Analyses** | | **All HOB Including CLABSI Analyses** | |
| --- | --- | --- | --- | --- | --- | --- |
|  | **Control** | **Cases** | **Control** | **Cases** | **Control** | **Cases** |
| N | 1745 | 349 | 1745 | 1172 | 1745 | 1521 |
| Length of stay | | | | | | |
| Adjusted^a^ mean days (95% CI) | 4.7  (4.2, 5.3) | 21.5  (19.3, 23.9) | 4.5  (4.1, 5.0) | 18.3  (16.6, 20.2) | 4.5  (4.1, 5.0) | 19.0  (17.3, 21.0) |
| Difference (case – control) | 16.8  *P* <0.001 | | 13.8  *P* <0.001 | | 14.5  *P* <0.001 | |
| Total hospital cost | | | | | | |
| Adjusted^a^ mean USD | $14,268  (11,325, 17,977) | $63,669  (50,026, 81,032) | $14,285  (13,481, 15,138) | $49,595  (46,835, 52,519) | $14,369  (11,464, 18,011) | $52,755  (42,062, 66,167) |
| Difference (case – control) | $49,400  *P* <0.001 | | $35,310  *P* <0.001 | | $38,386  *P* <0.001 | |
| Mortality rate | | | | | | |
| Unadjusted, n (%) | 134  (7.6%) | 109  (31.2%) | 134  (7.6%) | 320  (27.3%) | 134  (7.6%) | 429  (28.2%) |
| Adjusted %^a^ (95% CI) | 2%  (1, 5) | 9%  (4, 18) | 5%  (3, 7) | 15%  (10, 22) | 4%  (3, 7) | 15%  (10, 21) |
| Relative risk (cases vs controls) | 3.77  *P*<0.001 | | 3.20  *P*<0.001 | | 3.36  *P*<0.001 | |
| 30-day readmission rate | | | | | | |
| Unadjusted, n (%) | 232 (13.3%) | 60 (17.2%) | 232 (13.3%) | 190 (16.2%) | 232 (13.3%) | 250 (16.4%) |
| Adjusted %^a^ (95% CI) | 14%  (10, 19) | 19%  (13, 27) | 13%  (10, 18) | 17%  (13, 22) | 13%  (10, 17) | 18%  (14, 23) |
| Relative risk (cases vs controls) | 1.41  *P*=0.015 | | 1.28  *P*=0.009 | | 1.33  *P*=0.001 | |

^a^Adjusted for age, sex, ALaRMS value, and hospital-level variables (payer, staffed bed size, teaching status, and urban/rural location)

Note. ALaRMS, Acute Laboratory Risk of Mortality Score; BSI, bloodstream infection; CI, confidence interval; CLABSI, central-line associated bloodstream infection; HOB, hospital-onset bacteremia.
